# Prophage Excision in *Streptococcus pneumoniae* Serotype 19A ST320 Promote Colonization: Insight Into Its Evolution From the Ancestral Clone Taiwan 19F-14 (ST236)

**DOI:** 10.3389/fmicb.2019.00205

**Published:** 2019-02-08

**Authors:** Yi-Yin Chen, Jin-Town Wang, Tzu-Lung Lin, Yu-Nong Gong, Ting-Hsuan Li, Ya-Yu Huang, Yu-Chia Hsieh

**Affiliations:** ^1^Department of Pediatrics, Chang Gung Children’s Hospital, Chang Gung Memorial Hospital, College of Medicine, Chang Gung University, Taoyuan, Taiwan; ^2^Department of Microbiology, National Taiwan University College of Medicine, Taipei, Taiwan; ^3^Department of Internal Medicine, National Taiwan University Hospital, Taipei, Taiwan; ^4^Department of Medical Biotechnology and Laboratory Science, College of Medicine, Chang Gung University, Taoyuan, Taiwan; ^5^Research Center for Emerging Viral Infections, Chang Gung University, Taoyuan, Taiwan

**Keywords:** *Streptococcus pneumoniae*, mRNA sequencing, phage, adherence, *integrase*

## Abstract

*Streptococcus pneumoniae* 19A ST320, a multidrug-resistant strain with high disease severity that notoriously spread before the use of expanded pneumococcal conjugate vaccines, was derived from a capsular switching event between an international strain Taiwan 19F-14 (ST236) and a serotype 19A strain. However, the molecular mechanisms underlying the adaptive evolution of 19F ST236 to 19A ST320 are unknown. In this study, we compared 19A ST320 to its ancestral clone, 19F ST236, in terms of adherence to respiratory epithelial cells, whole transcriptome, and ability to colonize a young mouse model. Serotype 19A ST320 showed five-fold higher adherence to A549 cells than serotype 19F ST236. High-throughput mRNA sequencing identified a prophage region located between *dnaN* and *ychF* in both strains; however, the genes in this region were expressed at significantly higher levels in 19A ST320 than in 19F ST236. Analysis by polymerase chain reaction (PCR) showed that the prophage is able to spontaneously excise from the chromosome and form a circular episome in 19A ST320, but not in 19F ST236. Deletion of the *integrase* in the prophage of 19A ST320 decreased spontaneous excision and cell adherence, which were restored by complementation. Competition experiments in mice showed that the *integrase* mutant was six-fold less competitive than the 19A ST320 parent (competitive index [CI]: 0.16; *p* = 0.02). The 19A ST320 prophage-deleted strain did not change cell adherence capacity, whereas prophage integration strains (*integrase* mutant and 19F) had decreased expression of the down-stream *ychF* gene compared to that of 19A ST320. Further deletion of *ychF* significantly reduced cell adherence. In conclusions, these findings suggest that spontaneous prophage induction confers a competitive advantage to virulent pneumococci.

## Introduction

Prior to implementation of the 13-valent pneumococcal conjugate vaccine (PCV13 Prevnar 13, Pfizer), *Streptococcus pneumoniae* serotype 19A ST320 was prevalent in many countries (Choi et al., 2008; Moore et al., 2008; Ardanuy et al., 2009; Pillai et al., 2009; Shin et al., 2011; Hsieh et al., 2013). This clone emerged in South Korea before the introduction of seven-valent pneumococcal conjugate vaccine (PCV7) (Choi et al., 2008); surged in Taiwan, where only 20–30% of children < 5 years of age received ≥ 1 dose of PCV7 (Hsieh et al., 2013); and expanded as an important pathogen in the United States, Cananda, and Spain after the widespread use of PCV7 (Moore et al., 2008; Ardanuy et al., 2009; Pillai et al., 2009). *S. pneumoniae* serotype 19A ST320 is resistant to multiple antibiotics, and is a frequent cause of invasive pneumococcal disease, pneumonia, acute otitis media, and haemolytic uremic syndrome (Copelovitch and Kaplan, 2010; Kaplan et al., 2010; Greenberg et al., 2011). It was suggested that the serotype 19A ST320 strain was derived from a capsular switching event that occurred between an international strain, Taiwan 19F-14 (ST236), and a serotype 19A strain (Moore et al., 2008). Although ST320 is a double-locus variant of ST236, serotype 19A ST320 is more virulent than its ancestral clone Taiwan 19F-14 (ST236) in terms of invasive potential and the severity of pneumonia (Hsieh et al., 2009, 2011). In our previous study, we demonstrated that the genetic evolution from Taiwan 19F-14 (ST236) to 19A ST320 has made this pneumococcus better able to colonize of the nasopharynx on a mouse model without vaccine and antibiotic use (Hsieh et al., 2013). This evolution reflects not only a switch in capsular serotype but also, more importantly, changes in other loci. The genetic change from ST236 to ST320 conferred a significant competitive advantage in nasopharyngeal colonization. Although the complete genome sequence of the serotype 19A ST320 clone has been determined^1^, the basis for its superior colonizing ability compared to that of Taiwan 19F-14 (ST236) is unclear. In this study, we used whole transcriptomic analysis via high-throughput mRNA-sequencing to study the mechanism responsible for the difference in colonization effectiveness between the 19A ST320 and 19F ST236 clones.

## Materials and Methods

### Ethics Statement

Our animal procedures of this study were reviewed and approved by the Institutional Animal Care and Use Committee (IACUC) of Chang Gung University (Approval Number: CGU15-138), and the Committee recognizes that the proposed animal experiment follows the guideline as shown in the Guide for Laboratory Animal Facilities and Care as promulgated by the Council of Agriculture. Executive Yuan, ROC. BALB/cByJNarl mice were provided by National Laboratory Animal Center (NLAC), NARLabs, Taiwan. All animals were housed in an animal facility at 22°C, with a relative humidity of 55%, in a 12 h light/12 h dark cycle, with sterile tap water and food available *ad libitum*.

### Strains and Growth Conditions

Pneumococcal isolates form Chang Gung Children’s Hospital (CGCH) were grown at 37°C in Todd–Hewitt broth supplemented with 0.5% yeast extract (THY) or on blood agar supplemented with 5% defibrinated sheep blood in the presence of 5% CO_2_. *S. pneumoniae* is a biosafety level-2 microorganism. The experiments handling the bacteria should follow all appropriate guidelines and regulations. *Escherichia coli* was grown in Luria broth. Antibiotics were added at the following concentrations when requited: spectinomycin at 250 mg/L for *S. pneumoniae* and 100 mg/L for *E. coli* and chloramphenicol at 4 mg/L for *S. pneumoniae* and 30 mg/L for *E. coli*.

### Cell Adhesion Assays

A549 (ATCC CCL-185) human lung epithelial carcinoma cells were grown in Dulbecco’s modified Eagle’s medium (DMEM; Gibco) supplemented with 10% fetal bovine serum (FBS, Gibco) at 37°C in a humidified incubator at 5% CO_2_ and subcultured twice a week. Adhesion assays using A549 were performed as described previously (Nguyen et al., 2014), with some modification. Briefly, on the day before the adhesion assay, A549 cells were seeded in 24-well plates (1 × 10^5^ cells/well) and maintained in culture medium at 37°C in a 5% CO_2_ incubator. Mid-log phase (OD_600_ = 0.4 to 0.6) *S. pneumoniae* in THY were washed once with serum-free DMEM medium and then added to each well at a multiplicity of infection (MOI) of 100 and co-incubated for 2 h at 37°C in a 5% CO_2_ incubator. To determine the total numbers of adherent bacteria, the wells were washed three times with phosphate-buffered saline (PBS) to remove non-adhering bacteria, and 1 mL of deionized distilled water was added and incubated for 5 min to lyse cells. The adherent bacteria were enumerated by plating serial dilutions of the lysate on blood agar and counting the number of viable bacterial colony-forming units (CFUs). The experiments were performed in triplicate and repeated independently four times.

### Transcriptomic Analysis

For Illumina sequencing, total RNA was extracted from each sample (serotype 19A ST320 and Taiwan 19F-14 ST236) using the RNeasy mini kit (QIAGEN) and then treated with RNase-free DNase I (QIAGEN) for 15 min according to the manufacturer’s protocols. The integrity of the isolated total RNA was checked using an Agilent Technologies 2100 Bioanalyzer. Then, cDNA libraries were prepared according to the manufacturer’s instructions (Illumina), and an ABI StepOnePlus Real-Time PCR System was used for quantification and qualification of the sample library. The genome and gene information for serotype Taiwan 19F-14 ST236 and serotype 19A ST320 were downloaded from the NCBI database (accession numbers: CP000921.1 and CP001993.1, respectively). The expression level of each gene, as determined by RNA-Seq, was normalized to the number of reads per kilobase of exon region per million mapped reads (RPKM). The cut-off value for determining gene transcription was determined based on the 95% confidence interval for the RPKM values of each gene.

### Determination of mRNA Expression Levels by Quantitative Reverse Transcription Polymerase Chain Reaction (RT–qPCR)

An aliquot (400 ng) of total RNA from each bacterial strain was subjected to cDNA synthesis using SuperScript IV Reverse Transcriptase (Thermo Fisher). The cDNAs of *HMPREF0837_10263*, *HMPREF0837_10266*, *dnaN*, *ychF* and 23S rRNA were quantified using KAPA SYBR^®^ FAST qPCR Master Mix (KAPA Biosystems) and an ABI 7900 Real-Time PCR system. The cycling conditions for the quantitative real-time PCR (qPCR) were as follows: 50°C for 2 min and 95°C for 2 min, followed by 50 cycles of 95°C for 15 s and 60°C for 30 s, and after 50 cycles, melting curve analysis was performed from 60°C to 95°C, with a 2% ramp rate. Sequences of the primers used for RT–qPCR are listed in Table 1. The relative mRNA expression levels were calculated according to the ΔΔCt method, with normalization to 23S rRNA levels.

**Table 1:** Primers and plasmids used in this work.

Primer name	Sequence (5′ to 3′)	Purpose
int-up-F	ATG GAA CTC CAA CAG TAC GAT G	Δ*int*
int-dn-R	ATA TGA CAT GGT TAC TGC ACG A	
int-inverse-F	GGT TTT ACT CCT TTT TCC ATC	Δ*int* (iPCR)
int-inverse-R	TAA AAA AGT CCA TAA AAT TAT TAT TTC	
ychF-up-F	CAA AAC CTT TTC TAA TTG CTT G	Δ*ychF*
ychF-dn-R	ACA ATA CTA TCT TTG AAT GC	
ychF-inverse-F	TCT CCG TTT TCA TTT CAA TCC	Δ*ychF* (iPCR)
ychF-inverse-R	AAA TTA ATA AAT GGT GTC AAT TAG G	
dnaN-up-F	GTA AGA CTC ACT TAT TAA ACG	Δ*dnaN*
dnaN-dn-R	TTG CAA CTT TAG AAA GTG G	
dnaN-inverse-F	GGA TTC TCC TTT ATT TAT TTT TAG	Δ*dnaN* (iPCR)
dnaN-inverse-R	GTG AAA GAG GTT GAG CCT G	
Spec-F	GTG AGG AGG ATA TAT TTG AAT AC	Spectinomycin
Spec-R	TTA TAA TTT TTT TAA TCT GTT ATT TAA ATA GTT TAT AG	
Pint-F	AGA AAA TGA AGA AAA TTG TTT	Δ*int*::*int*
int-R	TTT TTC ATA AAA TGA AGT AGC	
Pcat-F	GTG ATA TAG ATT GAA AAG	Chloramphenicol
cat-R	TTA TTT ATT CAG CAA GTC	
12133-F	TTG CAA GAT AAG ATT ATC CAG	Δ*int*::*int*
12136-R	TTA AAC GGA TAT TCT TTA GAG	
12134-5-inverse-F	TAT TAA ACG ATA TAA GTT TG	Δ*int*::*int* (iPCR)
12134-5-inverse-R	CAA AAA ATA CGA AAA TGA TTA G	
10621-F	GAT TGA GAT CCA TTG AAACG	SP_10621
10621-R	AGA GAA CGT GTA ATC CCT GAA C	
C	ATG GAA CTC CAA CAG TAC GAT G	EP region
D	TCT TAT AAG TGT TCT GAA GC	
A	TGA AGA TGC TGG TTT GTT A	EC region
B	ATA TGA CAT GTT ACT GCA CGA	
RT-10621-F	CCC AGG GCT TGC AAA TTA CA	qPCR
RT-10621-R	CGA TCA TGT TGC CAG TTC GA	
RT-A	GCG ATA TGA TTT TGA GCG AAA A	qPCR
RT-B	CAA CGA TAC CTG CTG TCA AAG C	
RT-10263-F	CGT TCT TCA AAT GCT TCA TCG T	RT-qPCR
RT-10263-R	CGT ATC CGT GAC GGT TTC AA	
RT-10266-F	CCC CGT TTT CGC CTT GT	RT-qPCR
RT-10266-R	CCC CGT TTT CGC CTT GT	
RT-ychF-F	CGC GTG CAG CTT ACC ACTT	RT-qPCR
RT-ychF-R	CAA GCG CGA ACT TCT TTT TCA	
RT-dnaN-F	TCA GCT GTT CGT CCA TTT ACT CTT	RT-qPCR
RT-dnaN-R	TTA ATT TGT ACG AAC TGG TG	
RT-23S-F	ATG CGA CGA GCC GAC ATC	RT-qPCR
RT-23S-R	CCC CCA AGA GTT CAC ATC GA	

**Plasmid**	**Features**	**Reference**

pDL278	*spec*^R^; *E. Coli*-Streptococcous shuttle vector	(LeBlanc et al., 1992)
pKO3-Km	*cat*^R^ and *km*^R^; pKO3-derived plasmid	(Pan et al., 2008)
pGEM-T easy	T-A cloning vector	Promega
pJET1.2/blunt	Cloning vector	Thermo Fisher

### Determining the Population With Excised Prophage by Quantitative Real-Time PCR (qPCR)

The population with excised prophage were determined by qPCR. The following oligonucleotide pairs were designed for the experiment: prophage-excised-specific oligonucleotides to amplify a 151-bp fragment (RT-A/RT-B) near the *attB* site on the chromosome; non-prophage-specific oligonucleotides designed to amplify a 72-bp fragment (RT-10621-F/RT-10621-R) of *HMPREF0837_10621*. First, 19A ST320 genomic DNA was used as a template to amplify target gene fragments by PCR using the primer pairs 10621-F/10621-R (724-bp, SP_10621, for the non-prophage genomic region) and A/B (2,290-bp, EC, for the excised chromosomal DNA, “*attB*”), which were then cloned into pJET1.2/blunt (Thermo Fisher) to create plasmid standards for each target (named 10621/pJET and EC/pJET, respectively). Each amplification was carried out in a 20 μl reaction containing KAPA SYBR^®^ FAST qPCR Master Mix on an ABI 7900 system. Serial 10-fold dilutions of the plasmid standard containing 0.1–1 × 10^6^ copies were used to establish a standard curve to evaluate the Ct V.S. log_10_(copy number). To determine the changes in prophage excision over time, a culture of 19A ST320 was grown in THY at 37°C and 5% CO_2_ and genomic DNA was extracted at different time points. Then, the percentage of cells with excised prophage at each time point were analyzed by qPCR and normalized to the standard curve. To estimate the cell population with excised prophage at different time points, the number of copies of excised-chromosomal target region was divided by the number of copies of SP_10621 at each time point.

### Construction of Deletion Mutants

The *integrase* (*int*), *dnaN* and *ychF* gene-deletion strain (19A-Δ*int*, 19A-Δ*dnaN* and 19A-Δ*ychF*) was generated by replacing the gene with a spectinomycin resistance gene cassette as follow and primer used are listed in Table 1. Briefly, coding regions and flanking fragments for *int*, *dnaN* and *ychF* form the serotype 19A ST320 were amplified by PCR using primer pairs: int-up-F/int-dn-R for *int*, dnaN-up-F/dnaN-dn-R for *dnaN* and ychF-up-F/ychF-dn-R for *ychF*, respectively. The resulting PCR products were cloned into a pGEM^®^-T easy (Promega) plasmid. The coding regions of the *int*, *dnaN* and *ychF* were then removed by inverse PCR (iPCR) with primer pairs: int-inverse-F/int-inverse-R for Δ*int*, dnaN-inverse-F/dnaN-inverse-R for Δ*dnaN* and ychF-inverse-F/ychF-inverse-R for Δ*ychF*, respectively and ligated to a PCR amplified spectinomycin antibiotic cassette (spec-F/spec-R) form pDL278 plasmid to create a deletion construct vector. This vector was transformed into *S. pneumoniae* serotype 19A ST320 by Competence Stimulating Peptide-1 (CSP-1; GenScript), and transformants were selected on spectinomycin.

### Construction of an *integrase* Complementation Strain

To construct a complemented 19A ST320 *integrase* deletion mutant strain (19A-Δ*int*::*int*), a single copy of the *integrase* gene was inserted into the non-coding region between the *HMPREF0837_12134* and *HMPREF0837_12135* as follows. The *integrase* gene was PCR amplified from 19A ST320 by using the primer pair Pint-F/int-R, and then cloned into the pGEM^®^-T easy plasmid to generate P*int*::pGEM-T. A chloramphenicol (*cat*) antibiotic cassette from pKO3-Km plasmid was PCR amplified by using the primer pair Pcat-F/cat-R, and then sub-cloned into the SacII site of P*int*::pGEM-T to generate P*int*-*cat*::pGEM-T. Then, the primer pair Pint-F/cat-R was used to produce a P*int-cat* PCR fragment. Next, a 2,501-bp fragment of the insertion site was PCR amplified by using the primer pair 12133-F/12136-R and cloned into pGEM^®^-T easy to create p*12133-12136*::pGEM-T. A fragment generated by iPCR using the above plasmid and the primer pair 12134-5-inverse-F/12134-5-inverse-R was then ligated with the P*int-cat* PCR fragment to form the complementation vector p*12133*-P*int*-*cat-12136*::pGEM-T. The complementation strain (Δ*int*::*int*) was created by transforming the p*12133*-P*int-cat*-*12136*::pGEM-T plasmid into the 19A-Δ*int* strain using CSP-1 and selecting on chloramphenicol.

### *In vivo* Competition Assays

An *in vivo* competition assay for *S. pneumoniae* infection in mice was conducted as described previously (Hsieh et al., 2013). Mid-log phase (OD_600_ = 0.2–0.6) bacteria were combined at a 1:1 ratio (CFUs) in PBS, and the inoculating doses were confirmed by plating on blood agar plates. Then, 3-week-old female BALB/c mice were challenged intranasally (IN) with 50 μL of bacteria (containing 5 × 10^6^ CFUs/bacterial strain). After 7 days, the mice were sacrificed with CO_2_, and nasal lavages fluid was collected. The CFUs recovered were determined by serial dilution and plating on blood agar. For the competition experiment between wild-type strain and the *integrase* deletion mutant, the recovered bacteria were distinguished by replica plating on blood agar containing 250 mg/L spectinomycin. One hundred randomly picked colonies were tested per mouse. A competitive index (CI) was calculated based on the ratio of the competing bacterial strains recovered by nasal lavage, normalized to the ratio of the respective bacteria in the inoculum = (output_test strain_/output_wild type_)/(input_test strain_/input_wild type_).

### Statistical Analysis

Data are presented as means ± standard deviation (SD) from more than three independent experiments. Statistical significance was assessed by a two-tailed Student’s *t*-test. The mouse CI assay was analyzed by a one-sample Student’s *t*-test (one-tailed) using log-transformed CIs to determine if the indices were significant. A *p* value less than 0.05 was considered statistically significant. All statistical analyses were performed using SPSS 15.0 for Windows (Statistical Package for Social Sciences, Chicago, IL, United States).

## Results

### More Serotype 19A ST320 Cells Adhered to A549 Cells Than Serotype 19F ST236 Cells

**FIGURE 1:**
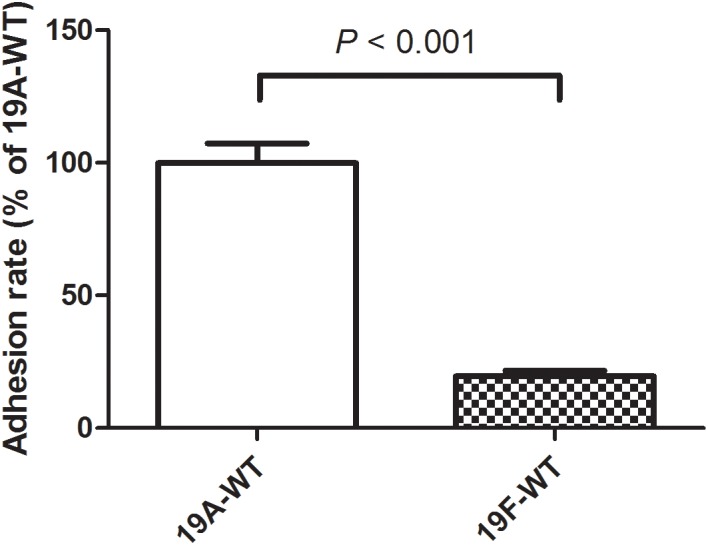
Adherence of serotype 19A ST320 wild type (19A-WT) and serotype 19F ST236 wild type (19F-WT) to A549 cells (*p* < 0.001, *n* = 4).

In a previous study, we demonstrated that the serotype 19A ST320 strain exhibited higher colonizing ability than its ancestral clone Taiwan 19F-14 (ST236) in mice (Hsieh et al., 2013). In this study, we demonstrated that the serotype 19A ST320 strain showed five-fold higher adherence to A549 cells than the serotype 19F ST236 strain (*p* < 0.001) (Figure 1).

### Transcriptomic Analysis

**FIGURE 2:**
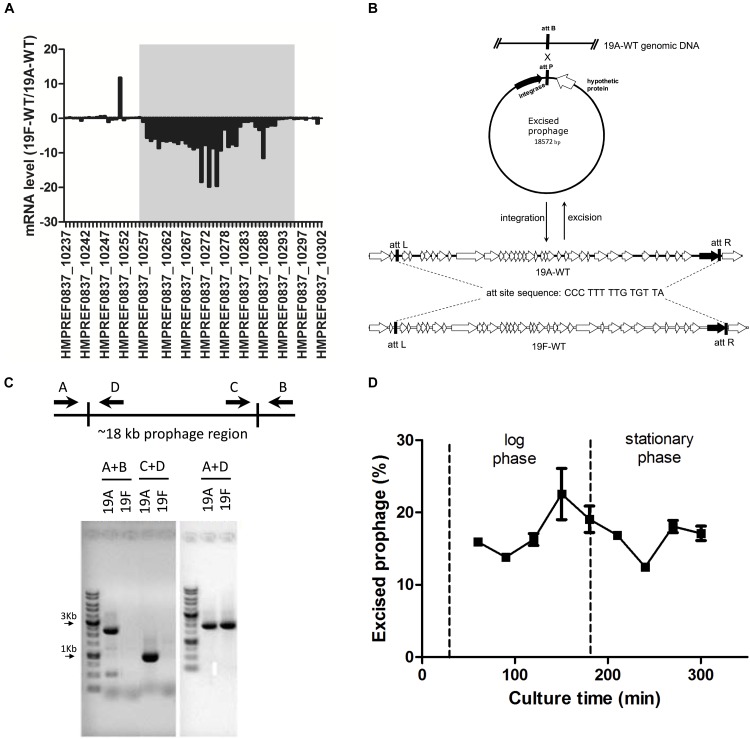
Transcriptomic analysis demonstrating spontaneous prophage induction in a serotype 19A ST320 strain. **(A)** The gray box shows the ratio (19F-WT/19A-WT) of the mRNA levels of genes in the prophage region of the *S. pneumoniae* serotype 19A ST320 genome. The mRNA expression levels of these genes were lower in 19F ST236 than in 19A ST320. **(B)** Gene organization of the prophage regions in 19F ST236 (19F-WT) and 19A ST320 (19A-WT) and a model of prophage excision in serotype 19A ST320. There is an additional transposase gene within the prophage region in 19F ST236. The putative *integrase* is shown as a black arrow. **(C)** PCR analysis for the presence of prophage excision and circular episomal prophage molecules. The relative positions of the primers on the chromosome are shown. Primer combinations A + B, which are located outside the prophage region, will amplify a product if the prophage is excised; otherwise, no PCR product will be amplified because this region is too long. Primer combination C + D, which are both located inside the prophage, will amplify a product if the prophage is excised and circularized. Primer combinations A + D, which are located inside and outside the prophage region, will amplify a product if the prophage is integrated in the chromosome. In serotype 19A ST320 (19A) strain, all three PCR products (A + B, A + D, and C + D) were detected; therefore, the prophage can excise and reintegrate spontaneously. In serotype 19F ST236 (19F), only A + D product was detected; therefore, the prophage is integrated in the chromosome. The PCR products were separated on 1% agarose gels and stained with ethidium bromide. **(D)** The percentage of the cell population with excised prophage and in serotype 19A ST320. When bacteria grew into late-log phase population of cells with excised prophage decreased. The average percentage of cells with excised prophage was 17.17 ± 3.19%.

The transcriptomic analysis revealed 801 genes that were differentially expressed between the serotype 19A ST320 and 19F ST236 strains, including 641 and 160 genes that were upregulated and downregulated in serotype 19A ST320, respectively (Supplementary Table S1 and Supplementary Figure S1). Of note, the mRNA levels of many genes located in the prophage region (∼18 kb) between *HMPREF0837_10256* (*dnaN*, encoding DNA polymerase III beta subunit) and *HMPREF0837_10294* (*ychF*, encoding a GTP-binding protein) were significantly higher in 19A ST320 than in 19F ST236 (Figure 2A). A RT-qPCR assay was then conducted on two genes within the prophage region (*HMPREF0837_10263* and *HMPREF0837_10266*) to verify the RNA-seq results. RT-qPCR confirmed an increase in the mRNA levels of these two genes in 19A ST320 compared to the corresponding levels in 19F ST236 (data not shown).

There are 34 open reading frames (ORFs) located within the prophage region in 19A ST320 and 19F ST236 (Figure 2B). But, there was an additional transposase gene in the prophage region in 19F ST236. A 14-bp direct repeat sequence “5′-CCC TTT TTG TGT TA-3′” flanked the prophage region, which should be the insertion site of this prophage. The GC content of the approximately 18 kb prophage region is 38%. These two strains shared 99% nucleotide identity in this prophage region.

### Demonstration of Epichromosomal Circular Prophage DNA in Serotype 19A ST320

We hypothesized that there was increased spontaneous prophage induction in 19A ST320, which accounted for the difference in mRNA levels of genes within the prophage between 19A ST320 and 19F ST236. PCR with several combinations of primers was used to assess spontaneous integration and excision of the prophage region in 19A ST320. If the prophage could excise from the bacterial chromosome to form an epichromosomal circular structure, the PCR products would be observed using primer pairs A/B (2,290-bp) and C/D (901-bp) (Figure 2C). PCR with the primer pair A/D (1,914-bp) would yield a product when the prophage is integrated into the bacterial chromosome (Figure 2C). In our experiment, we found that the prophage excise from the genome and exist as a double-stranded circular DNA molecule in the serotype 19A ST320 strain by PCR; whereas the prophage was integrated in the genome of the serotype 19F ST236 strain (Figure 2C). A DNA sequence analysis of the PCR fragments produced by primer pairs A/B and C/D confirmed the ends of the circular phage DNA were connected via one of the 14-bp direct repeat sequence (5′-CCC TTT TTG TGT TA-3′) flanking the prophage region (C/D); the other one 14-bp direct repeat was left in the chromosome (A/B) when the prophage region excised from the serotype 19A ST320 genome. We also tested for excision activity in 10 other serotype 19A ST320 isolates and 8 serotype 19F ST236 isolates by PCR. The results showed that all serotype 19A ST320 isolates had spontaneous prophage induction. Two of the eight serotype 19F ST236 isolates did not harbor the prophage, whereas the other six isolates had the prophage, but no spontaneous induction was observed (Table 2). Another 19 clinical isolates belonging to different serotypes were also examined. The results showed that these 19 clinical isolates did not contain the prophage (Table 2).

**Table 2:** The presence of prophage and prophage excision in clinical isolates of serotype 19A ST320, 19F ST236, and others serotypes.

Serotype	Numbers of isolates (n)	PCR products			Interpretation
			
		A + B	C + D	A + D	
19A	10	+	+	+	Prophage excision, integration
19F	6	−	−	+	Prophage integration, no excision
19F	2	+	−	−	No prophage
6B	3	+	−	−	No prophage
15A	2	+	−	−	No prophage
14	4	+	−	−	No prophage
23F	3	+	−	−	No prophage
15B	1	+	−	−	No prophage
3	6	+	−	−	No prophage

*+, PCR positive; −, PCR negative.*

### The Percentage of Cells in a Population With Excised Prophage

Bacterial cells were harvested at different growth phases, from early log phase to stationary phase, we found that the percentage of the cell population with excised prophage increased with cell growth into late-log phase, but decreased at stationary phase. The average percentage of cells with excised prophage across all culture time points was 17.17 ± 3.19% (Figure 2D).

### Spontaneous Prophage Induction Depends on *integrase* and Is Associated With Increased Adherence to Respiratory Epithelial Cells

**FIGURE 3:**
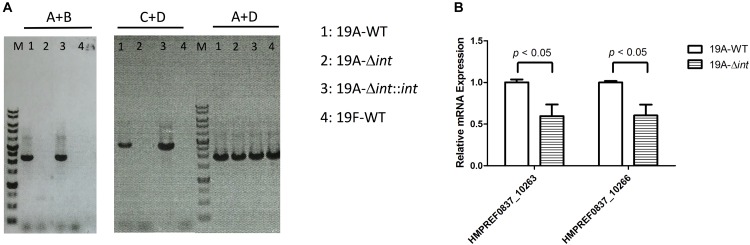
Integrase is responsible for prophage excision and higher expression of prophage genes in serotype 19A ST320. **(A)** PCR analysis for the presence of prophage excision and circular episomal prophage. PCR with primers A and B showed prophage excision in 19A ST320 wild type (19A-WT) and the *integrase* complementation strain (19A-Δ*int*::*int*). PCR with primers A and D showed prophage integration in all strains. PCR with primers C and D showed that there was circular prophage in 19A ST320 wild type (19A-WT) and the complementation strain (19A-Δ*int*::*int*). **(B)** The mRNA expression levels of *HMPREF0837_10263* and *HMPREF0837_10266* within the prophage region were significant decreased in the 19A ST320 *integrase* mutant (19A-Δ*int*) compared to those of 19A ST320 wild type (19A-WT). (*p* < 0.05, *n* = 3).

The prophage region of 19A ST320 harbors a putative *integrase* gene at the 3′ end (Figure 2B). The *integrase* gene was deleted to study whether it was responsible for prophage excision and integration. Following deletion of the *integrase* gene in 19A ST320, no prophage excision was detectable by PCR in 19A ST320 isogenic *integrase* mutant (19A-Δ*int*) (Figure 3A). Complementation of the deletion with an *integrase* gene at another chromosomal location (19A-Δ*int*::*int*) restored prophage excision (Figure 3A). Also, the mRNA expression level of genes (*HMPREF0837_10263* and *HMPREF0837_10266*) within the prophage region in the *integrase* deletion mutant (19A-Δ*int*) were significantly lower than that in 19A ST320 wild type (*p* < 0.05) (Figure 3B).

**FIGURE 4:**
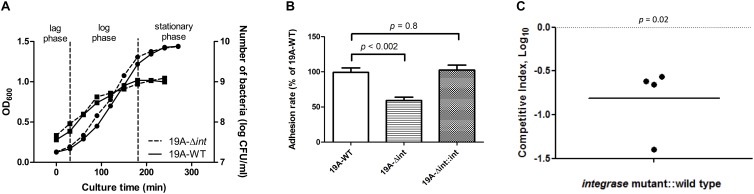
Integrase is associated with increased adherence and colonization. **(A)** Growth curve as measured by both OD_600_ (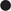, left *Y*-axis) and viable CFUs counts on blood agar (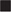, right *Y*-axis) of serotype 19A ST320 wild type (19A-WT) and its *integrase* deletion mutant (19A-Δ*int*). **(B)** Adherence of serotype 19A ST320 wild type (19A-WT), the *integrase* mutant (19A-Δ*int*), and the complementation strain (19A-Δ*int*::*int*) (wild type vs. mutant, *p* < 0.002, *n* = 4; wild type vs. complementation, *p* = 0.8, *n* = 4). **(C)** Intranasal challenge of 3-week-old female BALB/c mice with equal inocula of the bacterial strains. Each symbol represents the competitive index (CI) for an individual animal. A competitive index (CI) was calculated based on the ratio of the competing bacterial strains recovered by nasal lavage, normalized to the ratio of the respective bacteria in the inoculum = (output_test strain_/output_wild type_)/(input_test strain_/input_wild type_). The *integrase* mutant showed a lower CI compared to serotype 19A ST320 (*p* = 0.02, *n* = 4).

The growth curves and CFUs in different phases of the *integrase* deletion mutant and 19A ST320 wild type were similar (Figure 4A). We tested whether prophage excision is associated with adherence because there was a difference in adherence to respiratory epithelial cells between the serotype 19A ST320 and 19F ST236 strains (Figure 1). The isogenic *integrase* deletion mutant (19A-Δ*int*) of serotype 19A ST320 showed decreased adherence to A549 cells compared to the wild-type strain (*p* < 0.002), whereas the *integrase* complementation strain (19A-Δ*int*::*int*) showed restored cell adherence capacity that was similar to the wild-type strain (*p* = 0.8) (Figure 4B).

### Nasopharyngeal Colonization Competition in Young Mice

To reduce the variance caused by differences among mice, we performed a competition experiment in which mice were inoculated intra-nasal (IN) with equal CFUs of serotype 19A ST320 (19A-WT) and the isogenic *integrase* deletion mutant (19A-Δ*int*) to assess the role of prophage excision on colonization. At 7 days post-infection, the isogenic *integrase* deletion mutant (19A-Δ*int*) was sixfold less competitive than the parent serotype 19A ST320 strain in colonizing the mouse upper airway (CI = 0.16; *p* = 0.02; Figure 4C).

### Prophage Integration Decreased the Expression of the Downstream *ychF* Gene Which Was Associated With Cell Adherence

**FIGURE 5:**
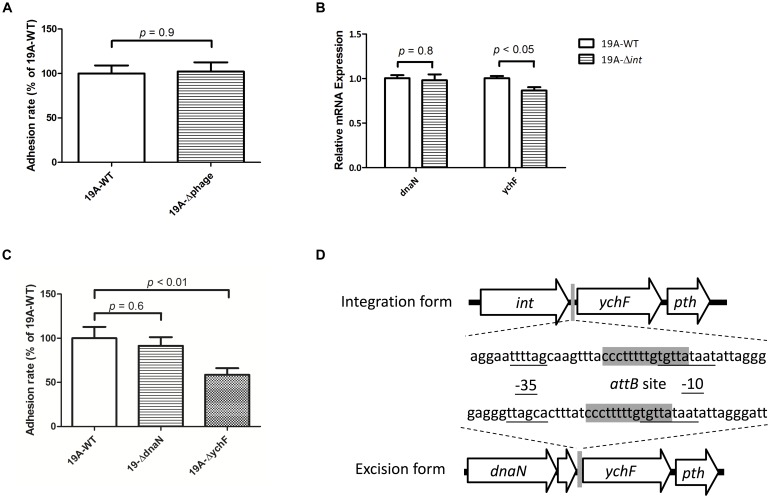
Circularization of prophage DNA affects the mRNA expression of the downstream *ychF* gene and cellular adhesion ability. **(A)** Deletion of the prophage region from 19A ST320 (19A-Δphage) did not affect the cell adherence ability, compared to the 19A ST320 wild type (19A-WT) (*p* = 0.9, *n* = 4). **(B)** The mRNA expression level of *ychF* in the 19A ST320 *integrase* deletion mutant (19A-Δ*int*) was lower than in the wild-type strain (19A-WT) (*p* < 0.05, *n* = 3). The mRNA expression level of *dnaN* was not different between the wild type and deletion mutant (*p* = 0.8, *n* = 3). **(C)** The cell adhesion ability of prophage downstream gene *ychF* deletion mutant (19A-Δ*ychF*) was decreased compared to the wild-type strain (*p* < 0.01, *n* = 4), but the adhesion ability of the prophage upstream gene *dnaN* deletion mutant (19A-Δ*dnaN*) was not (*p* = 0.6, *n* = 4). **(D)** Schematic showing that the promoter region sequence of *ychF* was rearranged and resulted in a different -35-promoter sequence when the prophage was excised from or integrated into chromosomal DNA of serotype 19A ST320. The -35-promoter sequence and -10-promoter sequence of *ychF* were predicted using the BPROM program (Softberry Inc., Mount Kisco, NY, United States; www.softberry.com). The 14-bp short sequence of the bacterial DNA *attB* site (5′-CCC TTT TTG TGT TA-3′) is shown in gray.

We created a 19A ST320 mutant devoid of the prophage region (19A-Δphage) to test whether the circular prophage DNA molecule directly modulates bacterial adherence. No difference was found in adhesion ability between the serotype 19A ST320 (19A-WT) and the prophage deletion mutant (19A-Δphage) (*p* = 0.9) (Figure 5A). This result indicated that the circular prophage, on its own, did not directly impact cell adherence. Next, we found that the mRNA expression level of *ychF* was different between strains 19A-WT and 19A-Δ*int* (*p* < 0.05) (Figure 5B). Transcriptome analysis also showed that the mRNA level of *ychF* was higher in serotype 19A ST320 than in 19F ST236. To analyze the bacterial adherence ability, as compared to the wild-type strain, we generated a 19A ST320 *ychF* deletion mutant (19A-Δ*ychF*). Deletion of *ychF* in the 19A ST320 strain did not impact growth (data not shown). However, 19A-Δ*ychF* had a lower bacterial adherence ability than 19A-WT (*p* < 0.01) (Figure 5C). The -35-promoter sequence of *ychF* is rearranged when the prophage region integrates into or excises from chromosomal DNA of serotype 19A ST320 (Figure 5D).

## Discussion

ST320 is the most predominant clone among 19A serotype *S. pneumoniae* isolates in both eastern and western countries (Beall et al., 2011; Shin et al., 2011). The high antibiotic resistance rates among ST320 strains are assumed to be one of the major reasons for its widespread dissemination and expansion. In response to antibiotic stress, *S. pneumoniae* shows a high rate of genetic transformation, which promotes the evolution of virulence (Prudhomme et al., 2006). In order to understand how the serotype 19A ST320 clone became an effective colonizer, we compared the serotype 19F ST236 clone to the serotype 19A ST320 clone and found that the mechanism of spontaneous prophage induction contributed to the competitive advantage of the serotype 19A ST320 clone. Through prophage excision and integration, the dynamic process act as a molecular switch that regulates the expression of *ychF* to increase bacterial adherence and drove the emergence of serotype 19A ST320 clone.

Bacteriophage, the most abundant biological entity on earth, play a crucial role in bacterial survival, activity and evolution (Rohwer and Edwards, 2002). Many bacterial genomes contain phage DNA, which may encode a virulence factor or increase bacterial fitness (Waldor, 1998). In fact, prophage acquisition has shaped the epidemiology of important bacterial pathogens (Banks et al., 2002). Residing in the ecological niche, the prophage is integrated into the bacterial chromosome of the serotype 19F ST236 clone, which decreased the expression of the downstream *ychF* gene. In serotype 19A ST320 clone, this prophage evolved to spontaneously excise from the chromosome, and this excision event was associated with increased expression of the downstream *ychF* gene, cell adherence and colonizing ability. Two direct 14-bp repeats flanking the prophage region (5′-CCC TTT TTG TGT TA-3′) were identified as the specific site of prophage integration. The gene encoding *integrase*, located at the 3′ junction of the prophage in serotype 19A ST320 stains, catalyzes the site-specific recombination between the prophage recognition site (*attP*) and a short sequence of bacterial DNA (*attB*) in serotype 19A ST320 strains. Several phages integrate their genome into the bacterial chromosome and exist in a lysogenic state, or replicate and lyse the bacterial host after induction. There are two lysogenic processes in which bacteria and phage interact: lysogenic conversion and active lysogeny. When the expression of phage-encoded proteins changes the bacterial phenotype and/or contributes to bacterial fitness, this is called lysogenic conversion, which is observed in *Corynebacterium diphtheria*, with the expression of diphtheria toxin; in *E. coli* O157:H7, with the expression of shiga toxins; and in *Vibrio cholera*, with the expression of cholera toxin (Freeman, 1951; Waldor and Mekalanos, 1996; Plunkett et al., 1999). In active lysogeny, an integrated prophage serves as a regulatory switch that controls the expression of bacterial genes, which is observed with the *comK* gene in *Listeria monocytogenes* and the mismatch repair system (MMR) in *Streptococcus pyogenes* (Rabinovich et al., 2012; Scott et al., 2012). Serotype 19F ST236 strains carry the prophage region, like serotype 19A ST320. Deletion of the prophage in serotype 19A ST320 did not change the cell adherence ability. The cooperative behavior between 19A ST320 and the prophage is likely to be active lysogeny. The prophage, upon integration into the genome of serotype 19A ST320, alters the expression of the *ychF* gene, probably via genome rearrangement of the promotor sequences. YchF, a conserved GTPase, has been shown to impact pneumococcal growth and virulence (Fernebro et al., 2008). In this study, we also found that YchF was involved in adherence to respiratory epithelial cells.

Despite numerous attempts, we could not isolate any phage particles from the culture medium of serotype 19A ST320 strains following UV irradiation and mitomycin C induction (data not shown). As we did not observe any phage structural genes, genes for DNA packaging, or the holing/lysin system for bacterial lysis, the prophage in the 19A ST320 strain did not seem to enter the lytic cycle and release new virions. However, the prophage has the replication protein and primase necessary for DNA replication. Increased mRNA expression of the prophage region in serotype 19A ST320 compared to serotype 19F ST236 is likely due to replication of the circular prophage genome as an autonomous element, just like the prophage CGP3 in *Corynebacterium glutamicum* and SF370.4 in *Streptococcus pyogenes* (Frunzke et al., 2008; Scott et al., 2008). The purpose of phage replication is to prevent its elimination from the bacterial population. In this study, we characterized the excisional activity of the 18kb prophage and evaluated its impact on pneumococcal adherence during colonization. However, the upstream events that lead to the activation of prophage excision and other phage-mediated mechanism relevant to colonization in serotype 19A ST320 strains remain unclear. Further studies are required to elucidate the mutually beneficial relationship between the bacteria and phages.

The availability and use of pneumococcal conjugate vaccines has dramatically decreased the incidence of invasive diseases caused by vaccine-targeted serotypes (Harboe et al., 2014). Nevertheless, the conjugate vaccines target only a small subset (up to 13) of the >90 known capsular serotypes of *S. pneumoniae*. This decline in the incidence of pneumococcal diseases caused by vaccine serotypes has been offset by substantial increases in both the carriage of and diseases caused by non-vaccine serotypes, which now occupy the niche vacated by vaccine-type pneumococci (Hicks et al., 2007; Singleton et al., 2007). Thus, vaccine-induced serotype replacement is gradually occurring (Chochua et al., 2017; Ladhani et al., 2018; Nakano et al., 2018). Moreover, certain successful lineages that were prevalent before the use of PCV, such as ST156, can persist after PCV implementation by undergoing capsular switching with a non-vaccine serotype (35B) (Chochua et al., 2017). The genome plasticity of pneumococci is high; therefore, it is an evolving threat to human health worldwide. Understanding the phage-related mechanisms that confer pneumococcal adaptation and pathogenicity offers a valuable perspective in our fight against pneumococcal disease.

## Author Contributions

Y-YC, J-TW, T-LL, and Y-CH designed the research, discussed the analysis, and revised the paper. Y-YC, T-HL, and Y-YH prepared materials and performed the experiments. Y-YC, T-LL, and Y-NG analyzed the data. Y-YC and Y-CH wrote the main text of the manuscript. All the authors reviewed and approved the final version of the manuscript.

## Conflict of Interest Statement

The authors declare that the research was conducted in the absence of any commercial or financial relationships that could be construed as a potential conflict of interest.

## Footnotes

1 http://www.ncbi.nlm.nih.gov/nuccore/CP001993.1
