# Supplementary material for: Prophage Excision in Streptococcus pneumoniae Serotype 19A ST320 Promote Colonization: Insight Into Its Evolution From the Ancestral Clone Taiwan 19F-14 (ST236)
Source: Front Microbiol. 2019 Feb 8;10:205. doi: 10.3389/fmicb.2019.00205 (PMC6375853; doi:10.3389/fmicb.2019.00205)
Supplement: Supplementary file 1 [file Data_Sheet_1.PDF]

Supplementary Material

Prophage excision in Streptococcus pneumoniae serotype 19A ST320 promote colonization: insight into its evolution from the ancestral clone Taiwan 19F-14 (ST236)

Yi-Yin Chen[1,6], Jin-Town Wang[2,3,6], Tzu-Lung Lin[4], Yu-Nong Gong[5], Ting-Hsuan Li[1], Ya-Yu Huang[1], Yu-Chia Hsieh[1]*

***Correspondence:**

Yu-Chia Hsieh
Department of Paediatrics, Linkou Chang Gung Memorial Hospital, No5, Fuxing Street, Guishan District, Taoyuan City 333, Taiwan.
E-mail addresses: yuchiahsieh@gmail.com
Telephone: +886-3-3281200 ext. 8231
Fax: +883-3-3286210

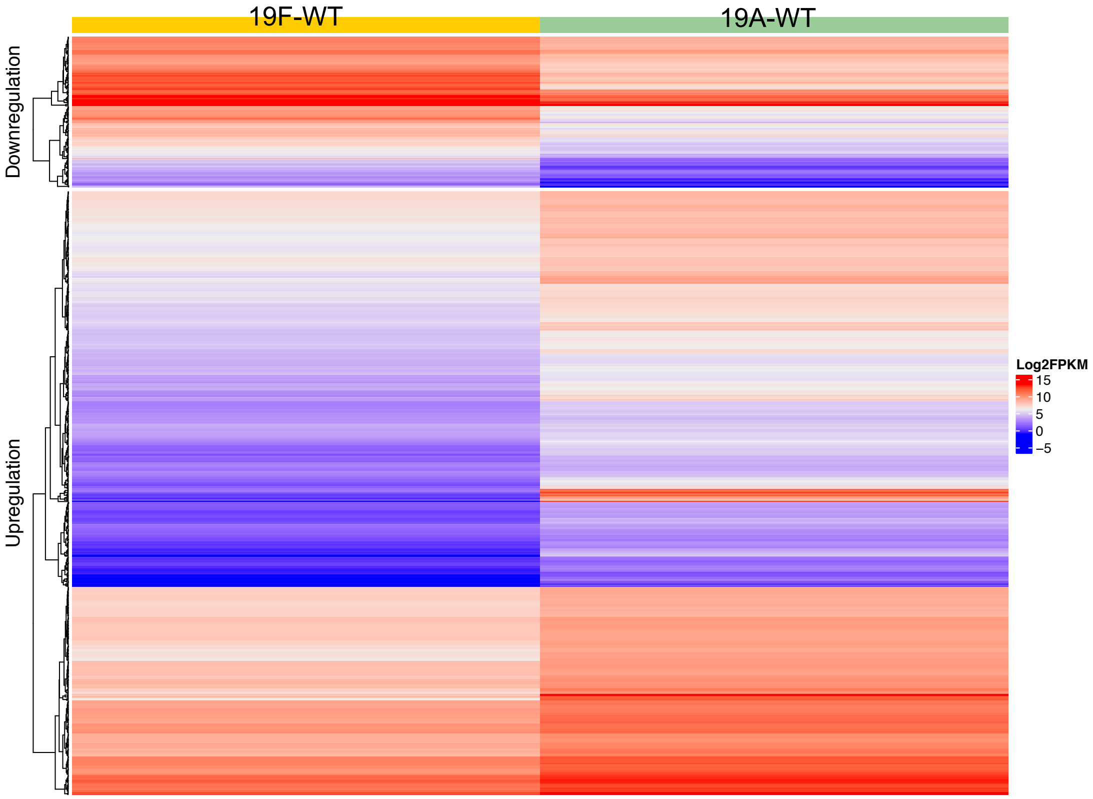

Supplementary Figure S1. Heat map and complete hierarchical clustering of differentially expressed mRNAs in 19A ST320 and 19F ST236.

Comparison of the transcriptomes of 19A ST320 (19A-WT) and19F ST236 (19F-WT) revealed 2467 differentially expressed genes. Expression is shown as the Log2 ratio of the fragments per kilobase of transcript per million mapped reads (FPKM). Comparing the gene expression in 19A-WT to 19F-WT, we identified 801 genes with a Log2 ratio fold change ≥ 1 or ≤ -1, including 641 upregulated genes and 160 downregulated genes. Heatmap and hierarchical cluster analysis based on FPKM values of 19F ST236 (19F-WT) and 19A ST320 (19A-WT) was performed using R version 3.4.2 and Complex Heatmap package version 1.10.2.
